# Improving the reference standard for the diagnosis of canine visceral leishmaniasis: a challenge for current and future tests

**DOI:** 10.1590/0074-02760180452

**Published:** 2019-01-31

**Authors:** Ana Izabel Passarella Teixeira, Debora Marcolino Silva, Tamires Vital, Nadjar Nitz, Bruna Caroline de Carvalho, Mariana Hecht, Diana Oliveira, Edward Oliveira, Ana Rabello, Gustavo Adolfo Sierra Romero

**Affiliations:** 1 Universidade de Brasília, Núcleo de Medicina Tropical, Brasília, DF, Brasil; 2 Universidade de Brasília, Faculdade de Medicina, Laboratório Interdisciplinar de Biociências, Brasília, DF, Brasil; 3 Grupo de Pesquisas Clínicas e Políticas Públicas em Doenças Infecciosas e Parasitárias, Belo Horizonte, MG, Brasil

**Keywords:** canine visceral leishmaniasis, diagnostic tests, serology

## Abstract

**BACKGROUND:**

Studies aimed at validating canine visceral leishmaniasis diagnostic tests present heterogeneous results regarding test accuracy, partly due to divergences in reference standards used and different infection evolution periods in animals.

**OBJECTIVE:**

This study aimed to evaluate the accuracy of the rapid test-dual path platform (TR-DPP) (Biomanguinhos®), EIE-Leishmaniose-Visceral-Canina-Biomanguinhos (EIE-LVC) (Biomanguinhos®), enzyme-linked immunosorbent assay (ELISA) rK39 (in-house), and the direct agglutination test (DAT-*Canis*) against a reference standard comprising parasitological and molecular techniques.

**METHODS:**

A phase II/III validation study was carried out in sample sera from 123 predominantly asymptomatic dogs living in an area endemic for visceral leishmaniasis.

**FINDINGS:**

Sixty-nine (56.1%) animals were considered infected according to the reference standard. For each test, the sensitivity and specificity, respectively, were as follows: TR-DPP, 21.74% [confidence interval (CI)95% 13.64% to 32.82%] and 92.59% (CI95% 82.45% to 97.08%); EIE-LVC, 11.59% (CI95% 5.9% to 21.25%) and 90.74% (CI95% 80.09% to 95.98%); ELISA rK39, 37.68% (CI95% 27.18% to 49.48%) and 83.33% (CI95% 71.26% to 90.98%); and DAT-*Canis*, 18.84% (CI95% 11.35% to 29.61%) and 96.30% (CI95% 87.46% to 98.98%).

**CONCLUSION:**

We concluded that improving the sensitivity of serum testing for diagnosing asymptomatic dogs must constitute a priority in the process of developing new diagnostic tests to be used in the visceral leishmaniasis control program in Brazil.

In the American continent, the control of metazoonotic canine visceral leishmaniasis (CVL) is crucial for reducing the occurrence of human visceral leishmaniasis (HVL); the dog is the main reservoir for *Leishmania* (*Leishmania*) *infantum*, the etiological agent of CVL in urban areas.^1^ ^,^ ^2^

The Brazilian Leishmaniasis Control Program prescribes detection and euthanasia for infected dogs, among other measures. The program sequentially uses two serological tests, which present a lower diagnostic accuracy in asymptomatic dogs or in those in the initial stages of the infection.^3^ ^,^ ^4^ ^,^ ^5^ ^,^ ^6^ ^,^ ^7^ ^,^ ^8^ Although some works indicate that serological tests may be used with efficacy for diagnosing asymptomatic animals,^6^ ^,^ ^9^ the performance of serological tests may be up to 20% inferior to the performance of molecular tests in that subgroup.^7^ ^,^ ^8^ ^,^ ^9^ ^,^ ^10^

Molecular diagnostic tests are not routinely used in public health programs because of the relative difficulties regarding the need for complex laboratorial infrastructure, the elevated costs, and the lack of commercially available assays, as well as other limitations.^7^ ^,^ ^8^ ^,^ ^9^ ^,^ ^10^

The diagnosis for animals with symptomatic CVL has fewer problems because those animals often present levels of antibodies that are specifically detectable through serological tests that are commercially available.^11^ ^,^ ^12^ ^,^ ^13^ Symptomatic animals also present a higher parasite load, allowing its detection in peripheral blood and tissues through molecular diagnosis methods.^14^

The role of infected dogs that remain asymptomatic in the transmission cycle of visceral leishmaniasis among dogs and from dogs to humans has not been widely clarified. Theoretically, a smaller parasite load in asymptomatic dogs would translate to a lower infectious capacity in sand flies. However, asymptomatic animals constitute most of the infected population, which means that they could be effectively relevant in transmitting the parasite to its vectors.^15^ ^,^ ^16^ ^,^ ^17^ ^,^ ^18^

Considering that most infected dogs remain asymptomatic for long periods, it is important that infected animals are accurately detected, regardless of the control measure to be applied to that canine population. In fact, mathematical modelling indicates that the key to the potential success of CVL control may be the identification of asymptomatic dogs.^19^ ^,^ ^20^

In that sense, new diagnostic tests are under development with promising results in their initial stages,^21^ ^,^ ^22^ ^,^ ^23^ ^,^ ^24^ but however, it is necessary to challenge these against a robust reference standard. The current context is characterised by a lack of consensus about the best reference standard for the validation of diagnostic tests for CVL, and few studies have used reasonably adequate reference standards.^5^ ^,^ ^25^

Among the tests under development, the DAT-*Canis* is based on the direct agglutination principle, which uses *Leishmania infantum* raw antigen. The test was developed by René-Rachou Institute (Fiocruz, Minas Gerais), and its prototype consists of a kit that allows up to 480 reactions at low costs (US$0.44/reaction, considering in-house testing), with a simple methodology of execution, and no need for complex laboratorial infrastructure.

The present research aimed to study the accuracy of certain tests against a reference standard constituted of a combination of parasitological and molecular techniques. The tests evaluated were as follows: rapid test-dual path platform (TR-DPP) (Biomanguinhos®); EIE-Leishmaniose-Visceral-Canina-Biomanguinhos (EIE-LVC) (Biomanguinhos®), which is currently recommended for CVL diagnosis in Brazil; the immunoenzymatic test using recombinant antigen rK39 (in-house); and the direct agglutination test (DAT-*Canis*) for serum samples from dogs resident in an endemic area of the Federal District of Brazil.

## MATERIALS AND METHODS

*Type of study* - This was a phase II/III validation study^26^ in serum samples collected consecutively in a random, non-preselected sample of dogs that reside in an endemic area for visceral leishmaniasis.

*Samples* - The samples used in this study are part of a biobank kept at the Laboratory for Leishmaniasis at the Centre for Tropical Medicine (NMT/FM/UnB) obtained during the study “Risk, diagnosis and prognosis of canine visceral leishmaniasis in the Federal District”. Samples were collected from dogs residing in an endemic area of the Federal District. They participated in the baseline assessment for a cohort study aiming to determine the role of socioeconomic factors and owner care on the risk of CVL acquisition. The dogs included in this study were randomly selected, and sample collection was done in the period between October 2015 and May 2017.

The criteria for sample inclusion in the present validation study were to have sufficient biological material available for carrying out serological testing, and to have taken all tests that composed the reference standard.

*Clinical assessment* - The dogs were clinically evaluated without previous knowledge of their infection status, and a signal score was attributed to each one according to a model adapted from that used by Proverbio et al.,^12^ which consisted of clinical aspects listed as items in a table with intensity degrees (Table I). The model used by us excluded the items that could not be determined through a single physical exam. The excluded items were appetite alterations, mental state alterations, intolerance to exercise, weight loss, polyuria, polydipsia, and proteinuria. These items, at their highest intensities, could have added up to 20 points to the score.

Since there was more than one veterinarian practitioner collecting samples and physical exams, other items, the assessment of which might have been subjective, were altered, such that only the presence or absence of these signals would be detected. These items were skin lesions, hepatosplenomegaly, epistaxis, vomiting, diarrhoea, claudication, altered pigmentation, hyperkeratosis, and onychogryphosis. This caused the exclusion of these items, which, at their highest intensities, could have added up to 21 points to the score.

Upon applying these adaptations to a pilot project with 20 assessments and two veterinarians, the modified clinical score could reach a maximum of 46 points instead of the 87 points obtained in the initial model.

*Composite reference standard* - The reference standard was composed by the following tests: amastigotes visualisation in the bone marrow smear; promastigotes isolation in bone marrow culture; parasite DNA detected by conventional polymerase chain reaction (PCR) targeting the conserved region of kDNA (and confirmed by PCR targeting the ITS1 gene) in peripheral blood and the bone marrow; and parasite DNA detected through the real time PCR technique (qPCR) targeting kDNA in the blood and the bone marrow.

All animals were submitted to all the tests composing the reference standard.

*Case definition* - The animal was considered infected when there was a positive result to any of the exams in the reference standard. The animal was considered not infected when there was a negative result in every test included in the reference standard.

*Execution of reference standard tests* - The professionals who executed the reference standard tests did not have knowledge of the characteristics of the animal that was the source of the samples.

Bone marrow smears were stained with Giemsa (Merck®) or Panótico (Instant Prov, Laboratory NewProv) for direct examination of the slides and observed in an optical microscope with an objective of 100x and immersion oil to identify amastigote forms.^27^ For each dog, at least two slides were examined.

A drop of aspirated bone marrow was inoculated into culture medium that was prepared according to the methodology adopted in the Leishmaniasis Laboratory in the Centre for Tropical Medicine of the University of Brasilia. Each sample was inoculated in duplicate, and the tubes were assessed every two days in an inverted optical microscope to seek typical movement that would indicate the presence of *Leishmania* spp. promastigotes during the 30-day period.^28^

*Molecular tests* - The conventional PCR method targeting kDNA^29^ was chosen for triaging the sample, and the ITS1 assay^30^ was used to confirm the presence of *Leishmania* spp. All precautions to avoid contamination were applied, including performing the pre-PCR, PCR, and post-PCR procedures in separate environments.

*DNA extraction* - After transport in refrigerated thermal boxes, the samples of blood mixed with EDTA were kept at -20ºC until processing. DNA was extracted using the Wizard Genomic DNA purification kit (Promega, Madison, WI, USA) according to the manufacturer’s instructions for a 300 µL sample. Lastly, the extracted DNA was hydrated overnight using 100 µL of hydration solution. For each extraction procedure, an ultra-pure water negative control was included.

**TABLE I:** Modified clinical score for canine visceral leishmaniasis (CVL) according to the adaptation from the model by Proverbio et al.^12^

Findings	Score			
	0	1	2	3
Bodily condition	Obese/Normal	Thin	Cachectic	--
Mucosae	Normal	Pale	Jaundiced	--
Dehydration	Absent	Light	Moderate to intense	--
Muscle atrophy on limbs	Absent	Light	Moderate to intense/widespread	--
Skin lesions	Absent	Present	--	--
Hepatosplenomegaly	Absent	Present	--	--
Conjunctivitis and / or Keratitis	Absent	Unilateral and light	Severe unilateral / bilateral	--
Uveitis and / or Blepharitis	Absent	Unilateral and light	Severe unilateral / bilateral	--
Lymph adenomegaly	Absent	1 to 2 lymph nodes	--	3 or more / widespread
Epistaxis	Absent	Present	--	--
Mouth ulcers or nodules	Absent	1 to 2	3 or more	--
Vomit	Absent	Present	--	Frequent, with vomit
Diarrhea	Absent	Present	--	--
Claudication	Absent	Present	--	--
Erythema	Absent	1 to 25% of the body	25 to 40% of the body	40% or more of the body
Dry exfoliative dermatitis	Absent	1 to 25% of the body	25 to 40% of the body	40% or more of the body
Ulcerative dermatitis	Absent	1 to 25% of the body	25 to 40% of the body	40% or more of the body
Nodular dermatitis	Absent	1 to 25% of the body	25 to 40% of the body	40% or more of the body
Pustular dermatitis	Absent	1 to 25% of the body	25 to 40% of the body	40% or more of the body
Alopecia	Absent	1 to 25% of the body	25 to 40% of the body	40% or more of the body
Altered pigmentation	Absent	Present	--	--
Hyperkeratosis of truffles and cushions	Absent	Present	--	--
Onychogryphosis	Absent	Present	--	--

*kDNA-conserved region PCR* - The reaction was carried out using one forward: 5’GGG GAG GGG CGT TCT GCG AA 3’ and two backward: BW-CA: 5’CCG CCC CTA TTT TAC ACC AAC CCC 3’ and BW-B: 5’GGC CCA CTA TAT TAC ACC AAC CCC 3’ targeting 120 bp of the kDNA-conserved region.^29^ Genomic DNA samples extracted from *Leishmania infantum* (MCER/BR/79/M6445), *L.* (*L.*) *amazonensis* (MHOM/BR/75/M2904), and *L.* (*Viannia*) *braziliensis* (IFLA/BR/67/PH8) were used as positive controls, and the same negative control was used as in the extraction step.

A 10 µL reaction was done with 1 µL of DNA sample, 0.3 µL of Taq (5 U), 1 µL of dNTPs (0.5 mM), 0.5 µL initiators BW-B and BW-CA (in the concentration of 10 µM) and 1.0 µL FW (10 µM), 1 µL of MgCl_2_ (50 mM) and 1 µL of 5X buffer. The reaction was carried out in a Techne FTC-PLUS thermocycler (Bibby Scientific LTD, United Kingdom) with the following settings: initial denaturalisation of 95ºC for 5 min, 35 cycles (heating at 95ºC for 30 s, annealing at 64ºC for 30 s and extension at 72ºC for 30 s), and final extension at 72ºC for 5 min.

*ITS1 gene PCR* - The ITS reaction was carried out using ITS-219F primers (5’ AGC TGG ATC ATT TTC CGA TG 3’) and ITS-219R (5’ ATC GCG ACA CGT TAT GTG AG 3’); the size of the amplicon was 265 bp.^30^ For that group of PCR reactions, the positive control was DNA extracted from a *L. infantum* promastigote culture (MCER/BR/79/M6445). For the negative control, MilliQ water was used. The reaction was done in a total volume of 25 µL, with 3 µL DNA, 0.5 µL of each primer (10 µM), 0.2 µL of dNTPs (0.5 mM), 5.0 µL of buffer 5X, 0.3 µL of Taq (5U), and 0.75 µL of MgCl_2_ (50 mM). The reaction was performed in a thermocycler (Techne FTC-PLUS Bibby Scientific LTD, United Kingdom) using the following cycling conditions: initial denaturation at 95ºC for 5 min; 40 cycles of heating at 95ºC for 30 s, annealing at 57ºC for 30 s, and extension at 72ºC for 30 s; and final extension at 72ºC for 5 min. The PCR products were maintained at 4ºC until viewed in a 7.5% polyacrylamide gel (150 volts, 75 Amp for 90 min) followed by silver staining.

*Real-time PCR (qPCR)* - The reaction was set off with the primers 5’ GGC CCA CTA TATTAC ACC AAC CCC 3’ and 5’ GGG GTA GGG GCG TTC TGC GAA 3’, as described by Pita-Pereira et al.^31^ Each reaction was carried out using 0.2 µM of each initiator, 2 µL of DNA samples (25 ng/µL), and 1X Power SYBR Green PCR Master Mix(2 µL), with the final volume per reaction being 20 µL. All reactions were conducted using two positive controls containing 5 ng/µL and 0.005 ng/µL of DNA extracted from the *L. infantum* promastigotes (MCER/BR/79/M6445). Two negative controls were also set: a blank without DNA and another control with DNA from HEK cell culture. The results were considered positive up to Ct 35, and showed a sensitivity of 0.005 ng/mL for *L. infantum* DNA. The results were classified as positive or negative. The thermocycler QuantStudio 3 (Applied Biosystems) was used to perform qPCR, with the following cycling conditions: initial denaturation at 94ºC for 12 min and 40 cycles of denaturation at 94ºC for 30 s, annealing at 55ºC for 30 s, and extension at 72ºC for 8 s.

*Serological tests* - The TR-DPP^®^ Leishmaniose Visceral Canina (Biomanguinhos, Rio de Janeiro, RJ, BR) and ELISA (EIE-Leishmaniose Visceral Canine) (Biomanguinhos®) tests were carried out in serum samples sent to the Environment Surveillance Laboratory of the Federal District (DIVAL), the government branch that is responsible for controlling zoonoses in the Federal District. The tests were executed according to manufacturer’s recommendations and to parameters established by the Ministry of Health.^32^

The ELISA rK39 was carried out in the René-Rachou, Fiocruz, Research Institute in Belo Horizonte. The Nunc MaxiSorp plates were sensitised with a rK39 antigen at a concentration of 1 µg/mL diluted in carbonate/bicarbonate (pH 9.6) in a wet chamber inside a refrigerator (2 to 8ºC) overnight. Afterwards, they were frozen at -20ºC until use. The plates were washed three times with a phosphate buffer containing Tween 0.05% (TF-Tween). Additionally, 200 µL/well of diluted 5% milk (TF-Tween-milk 5%) was added to each plate well and incubated for 2 h in a wet chamber at 37ºC. After new lavages, the plates were incubated in a wet chamber at 37ºC with 50 µL/well of anti-IgG canine conjugate with peroxidase (Sigma-Aldrich, St. Louis, MO, USA) diluted 1:50.000 in 1% TF-Tween-Milk.

The plates were washed again and incubated for 5 min in the dark with TMB (tetramethylbenzidine, Sigma-Aldrich) at 50 µL/well, and the reaction was blocked with 50 µL/well of a solution of 1 N sulfuric acid. The reading was carried out using the FlexStation 3 Multi-Mode Microplate Reader (Molecular Device, San Jose, CA, USA) with a wavelength of 450/620 nm. The cut-off was defined by the average of absorbance readings in 13 negative control samples increased by two standard deviations. The samples that presented a difference in absorbance readings over 20% were retested.

*DAT-Canis* - The test kit used for serum agglutination, DAT-*Canis*, is composed of 10 lyophilised antigen vials containing stained promastigotes of *L. infantum* (MHOM/BR/2002/LPC-RPV), a rehydration solution vial, and a sample diluent vial.

First, the lyophilised antigen was rehydrated with 5 mL of rehydration solution. A total of 99 µL of sample diluent was deposited in the well in the first V-bottom plate column (Greiner BioOne Produtos Médicos Hospitalares, Americana, SP, BR) and 50 µL in the remaining ones (2nd to 4th). Next, 1 µL of the serum samples was diluted in the well from the first column, making a dilution of 1:100. Then the samples were diluted to 1:800. The plates were incubated for an hour at room temperature and 50 µL of the previously rehydrated antigen was added. The plates were agitated for 5 min and incubated for 18 h in a dark chamber. Positive and negative control sera were tested in parallel with each plate. The results were visually read with the naked eye. The cut-off adopted for defining positive and negative results was 1:400, as previously described.^21^ The positive samples from the qualitative test were retested in the same manner and diluted until they reached the proportion of 1:102,400.

**Fig. 1:**
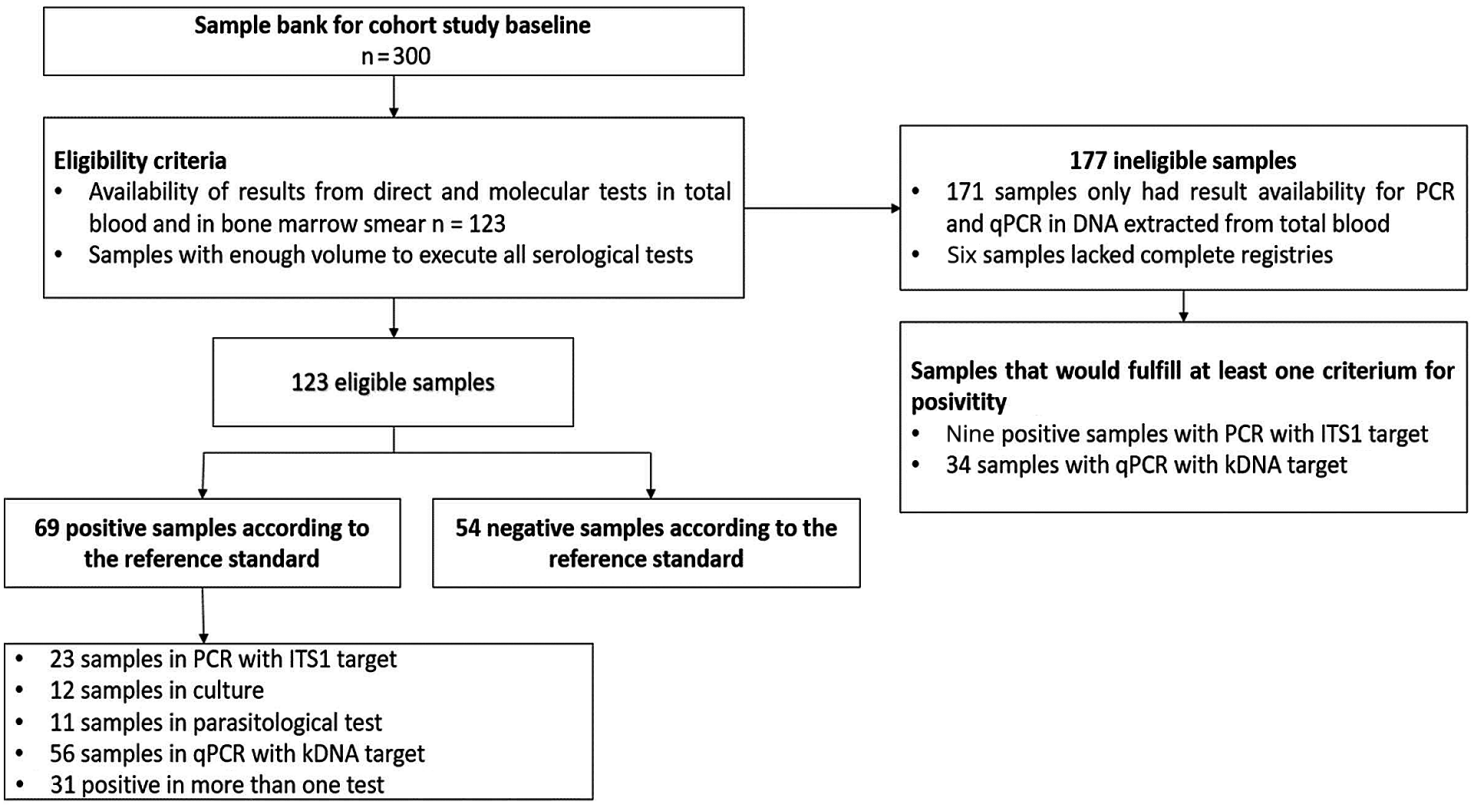
sample selection flowchart.

*Statistical analysis* - The test results and information on the dogs that were the sources of the samples were tabulated in Microsoft Excel. The Statistical Package for the Social Sciences (SPSS, IBM, Armond, New York, United States) was used to calculate the accuracy parameters and their respective 95% confidence intervals. The accuracy was calculated with the sequential use of diagnostic testing in different combinations. The positive and negative predictive values were calculated along with the positive and negative likelihood ratios. A sensitivity analysis was done regarding the positive and negative predictive values for a hypothetical spectrum of infection prevalence values. Also, a comparison of the clinical score between infected and non-infected animals was performed with the Mann-Whitney U test.

*Ethics* - The collection of biological samples for the study was approved by the Ethics Committee on Animal Use (CEUA, from the acronym in Brazilian Portuguese) at the Institute of Biological Sciences at the University of Brasília, under UnBDoc number 11253/2015.

Legally competent owners voluntarily consented to the participation of their animals in the study, and they signed consent forms.

## RESULTS

Sixty-nine out of the 123 animals (56.09%) were considered infected according to the composite reference standard. From the animals that presented positive results in the reference standard, 36.23% (25/69) were positive during the screening with kDNA PCR and confirmed through ITS1 PCR; 17.39% (12/69) had positive culture and direct parasitological test; and 81.15% (56/69) were positive in the kDNA qPCR. The raw agreement among the tests that composed the reference standard is described in Supplementary data (Table I). Fig. 1 introduces the sample selection process.

*Characteristics of dogs* - All 123 dogs were housed in the Federal District, with 77 (62.6%) in the administrative area of Fercal, 39 (31.7%) from Sobradinho II, four (3.25%) from Lago Sul, and three (2.44%) from Plano Piloto. The referred average age of the dogs was 3.21 years (DP = 2.91 years). Sixty-two (50.41%) were male, 59 (47.97%) were female, and two animal registers did not include that information.

**Fig. 2:**
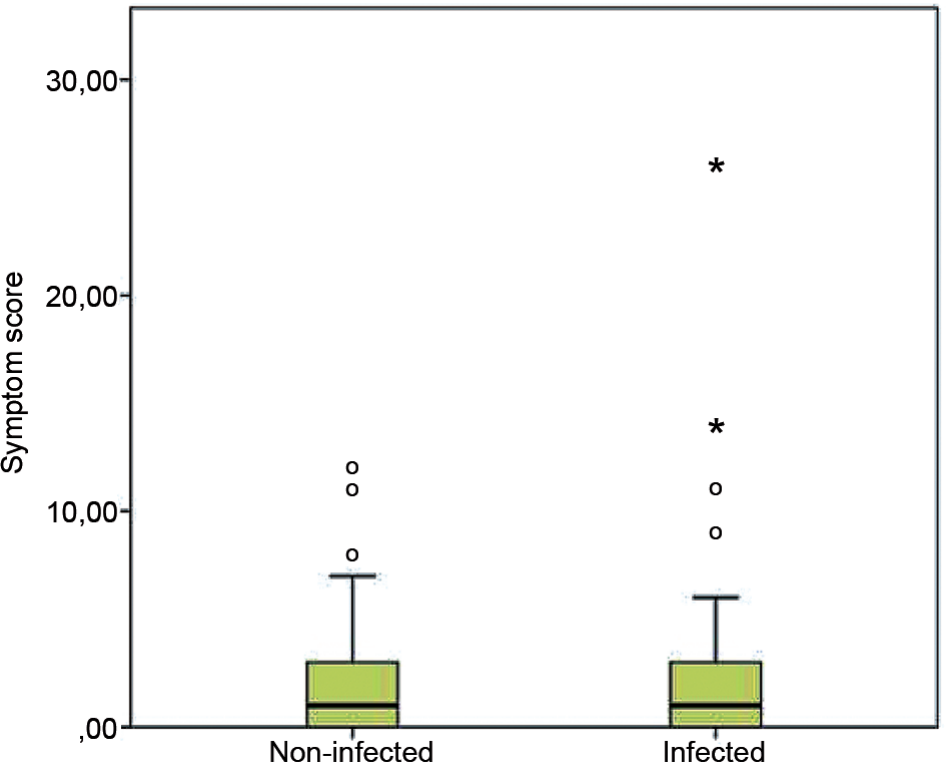
distribution of clinical score for canine visceral leishmaniasis (CVL) in groups of infected and non-infected animals. Fifty-five non-infected dogs, median 1 (IQR:0-3; interval 0-12 points). Sixty-six infected dogs, median 1 (IQR:0-3; interval 0-26 points). (Mann-Whitney U test; p = 0.708).

Most of the animals presented few or no CVL symptoms. The average score for clinical signs suggestive of CVL was 2.13 points (DP = 3.44 points; median = 1.0 point; quartiles 25 and 75 of 0 and 3 points, respectively). The dogs that were not infected and were completely asymptomatic (zero score) were 22/55 (40%), and the infected and asymptomatic dogs were 24/66 (36.36%). Out of the 55 non-infected dogs, 46 (83.64%) presented a score equal to or lower than 3, whereas 55 out of the 66 infected dogs (83.33%) presented a score equal to or lower than 3, as shown in Fig. 2.

*Accuracy of serological tests* - The positivity observed in serological tests was 15.4% (19/123) in TR-DPP, 10.6% (13/123) in EIE-LVC, 28.5% (35/123) in ELISA rK39, and 12.2% (15/123) in DAT-*Canis*. The raw agreement among the serological tests is demonstrated in Supplementary data (Table II).

Table II presents the frequency of results in serological tests in relation to the reference standard and its accuracy values.

Several sequential combinations among the serological tests were evaluated. The specificity nearly always reached 100%.

The protocol recommended by the Ministry of Health, which used TR-DPP as screening test, would detect 19 samples as positive, and the EIE-LVC would confirm seven out of these 19 samples as positive. The combined sensitivity (7/69) would be 10.14% (CI95% 5.0 to 19.49), and the combined specificity (54/54) would be 100% (CI95% 93.36 to 100).

The combination of TR-DPP as screening tests followed by DAT-*Canis* as a confirmatory test would repeat the results from the Ministry protocol. However, if ELISA rK39 were the confirmatory test, out of the 19 positive samples from the screening, nine would be confirmed as positive. Thus, the sensitivity and specificity of that last combination would be 9/69 or 13.04% (CI95% 7.02 to 22.97) and 54/54 or 100% (CI95% 93.36 to 100), respectively.

**TABLE II:** Accuracy of serological tests for canine visceral leishmaniasis (CVL), Brasília, DF, 2017

Index tests		Reference standard		Test accuracy (%)		Likelihood ratio	
		Positive (+)	Negative (-)	Sensitivity (CI95%)	Specificity (CI95%)	Positive (CI95%)	Negative (CI95%)
DAT-*Canis* ^*a*^	(+)	13	2	18.84	96.30	5.08	0.84
	(-)	56	52	(11.35 to 29.61)	(87.46 to 98.98)	(1.19 to 21.58)	(0.74 to 0.95)
EIE -LVC^*b*^	(+)	8	5	11.59	90.74	1.25	0.97
	(*-*)	61	49	(5.99 to 21.25)	(80.09 to 95.98)	(0.43 to 3.61)	(0.86 to 1.09)
R-DPP^*c*^	(+)	15	4	21.74	92.59	2.93	0.84
	(-)	54	50	(13.64 to 32.82)	(82.45 to 97.08)	(1.03 to 8.33)	(0.73 to 0.97)
ELISA rK39^*d*^	(+)	26	9	37.68	83.33	2.26	0.74
	(-)	43	45	(27.18 to 49.48)	(71.26 to 90.98)	(1.15 to 4.41)	(0.60 to 0.93)

a: direct agluttination test; b: immuno-enzymatic assay for CVL; c: rapid test dual path platform; d: enzyme linked immunosorbent assay rk39.w

With DAT-*Canis* as a screening test, out of the 15 positive samples, seven would be confirmed by TR-DPP with a combined sensitivity of 7/69 or 10.14% (CI95% 5.0 to 19.49) and the combined specificity of 54/54 or 100% (CI95% 93.36 to 100).

Using DAT-*Canis* for screening and ELISA rK39 for confirmation, out of the 15 CVL positive samples, nine would be confirmed as being infected; their combined sensitivity would be 9/69 or 13.04% (CI95% 7.02 to 22.97), and their combined specificity would be 53/54 or 98.15% (CI95% 93.36 to 100). Supplementary data (Table III) provides further information on the accuracy figures for combined serological tests.

In the 56% prevalence of infection scenario observed in the actual sample of dogs, the positive predictive value (PPV) and negative predictive value (NPV) for the tests were as follows: DAT-*Canis*, 0.88 and 0.49; TR-DPP, 0.79 and 0.49; ELISA rK39, 0.74 and 0.53; and EIE-LVC, 0.60 and 0.55. The sensitivity analysis for PPV and NPV for a hypothetical scenario of infection prevalence between 0 and 70% demonstrated similar behaviour between the tests, as shown in Supplementary data (Figure).

## DISCUSSION

The diagnostic protocol using TR-DPP as the screening test and EIE-LVC as the confirmatory test for CVL has been implemented in Brazil since 2012.^33^ Studies conducted later have evaluated the sensitivity and specificity of these tests.^4^ ^,^ ^5^ ^,^ ^8^ ^,^ ^34^ ^,^ ^35^ ^,^ ^36^ ^,^ ^37^ However, the lack of a homogenous reference standard has caused the results of these studies to diverge, as each one has used a different criterion for defining infected cases.

With a reference standard in which dogs were considered negative when they had negative results in other serological tests for CVL and positive by the direct parasitological test in bone marrow smear, TR-DPP had a sensitivity of 98% in symptomatic dogs.^35^ In another study with asymptomatic and symptomatic dogs that used the immunohistochemical examination of skin biopsies as a reference, the accuracy for TR-DPP reached sensitivity levels of 82.1% (CI95% 73.7 to 88.4) and a specificity of 98.9% (CI95% 94.4 to 99.8).^34^ Another study that used as a reference standard a combination of parasitological tests, including parasite cultures, conventional H&E histopathology, and immunohistochemical examination to assess TR-DPP showed a sensitivity of 88.0% (CI95% 67.5 to 96.8) and specificity of 69.2% (CI95% 63.7 to 74.3).^4^ A larger study, using the same reference standard, made subgroup analysis based on the presence of symptoms revealing that TR-DPP for asymptomatic dogs had a sensitivity of 75% (CI95% 42.8 to 94.5) and specificity of 72.9% (CI95% 68.5 to 77.1) compared to sensitivity of 93.8% (CI95% 82.8 to 98.7) and specificity of 56.4% (CI95% 46.2% to 66.3%) in symptomatic dogs.^8^ Finally, DAT-*Canis* showed a sensitivity of 97% in symptomatic dogs,^21^ and its performance had not been previously assessed in diagnosing asymptomatic dogs.

Peixoto et al.^5^ performed a meta-analysis of test accuracy for CVL diagnosis, and they estimated a sensitivity of 83.5% (CI95% 78.3 to 87.9) and a specificity of 72.9% (CI95% 70.5 to 75.2) for TR-DPP and a sensitivity of 89% (CI95% 86.9 a 90.9) and specificity of 87% (CI95% 85.6 to 88.3) for the ELISA immunoenzymatic test with raw antigens. In that review, the heterogeneity was identified in the type of reference standard used in the studies that were included in the meta-analysis. The scarcity of studies with an asymptomatic dog population was also evident.^5^

A study that used only the parasitological diagnosis as a reference estimated a sensitivity of 46.2% for TR-DPP and of 46.3% for EIE-LVC.^37^ Another more recent study used the direct parasitological test associated with the parasite culture, and it estimated sensitivity for TR-DPP of 100%; its specificity varied between 22 and 96%.^38^ The study by Silva et al.^39^ used serological tests as a reference standard and the TR-DPP’s sensitivity was 58.33% (CI95% 43 to 72). Carvalho et al.^10^ demonstrated that by using qPCR in blood serum samples, the protocol for CVL diagnosis prescribed by the Ministry of Health had a sensitivity of 67.24% (CI95% 54.42 to 77.92) and specificity of 86.59% (CI95% 77.55 to 92.34).

Nevertheless, it is valid to highlight that because of its higher sensitivity and lower costs, some researchers recommend EIE-LVC as a screening test and TR-DPP, due to its higher specificity, as a confirmatory test in the protocol for CVL diagnosis in Brazil.^34^ ^,^ ^36^ These recommendations are consistent with the results from the present study in which the sensitivity was higher when tests were analysed in that sequence.

In the context of the imperfection and heterogeneity of the tests used as a reference standard in the validation studies, an alternative would be the use of latent-class analysis to estimate indirectly the accuracy of tests, as Solcà et al.^40^ did. In that study, the results for sensitivity for TR-DPP and EIE-LVC were 47.1% and 43.8%, respectively. Their data are substantially different from the accuracy studies that used other approaches for validation.^40^

The difficulty in establishing a reference standard also resides in the lack of consensus on how to determine whether a dog is infected. Traditionally, serological tests were used as a reference to define the state of infection; however, there was the acknowledged lack of sensitivity. More recently, molecular techniques have allowed the detection of infection in animals that are seronegative.^14^ ^,^ ^17^ This more sensitive form of detecting asymptomatic infection can contribute to better understanding of the dynamics of transmission and the lack of efficacy in the measures for CVL control that are currently based on the application of serological tests. That is a relevant point because these dogs, although asymptomatic, may act as competent reservoirs.^15^ ^,^ ^16^ ^,^ ^17^ ^,^ ^18^ ^,^ ^19^ ^,^ ^20^ ^,^ ^41^

Similarly, the diagnosis of asymptomatic infection in humans is also challenging because the time between infection development and production of antibodies at detectable levels is not clear, providing room for molecular techniques for enhancing the early detection of infection.^42^ ^,^ ^43^

In a context in which the absence of a well-defined reference standard interferes with study results, this is the first study to use a composite reference standard that includes both conventional parasitological techniques and a combination of molecular tests in blood and bone marrow samples that can be obtained with relative ease.

With this composite reference standard, the accuracy results for the DAT-*Canis* test were similar to the those of other evaluated tests that have been used for routine CVL diagnosis in the visceral leishmaniasis control program in Brazil. Despite this similarity, the results for the accuracy values were inferior to those observed in previous studies.^5^ ^,^ ^8^ ^,^ ^21^ ^,^ ^44^ The main difference between the present study and previous studies is at least partly caused by the type of reference standard used in each study. In this study, the introduction of very sensitive molecular tests applied to blood and bone marrow samples certainly contributed to the identification of 56.09% of the samples as positive. If the reference standard had simply involved the direct parasitological examination and culture of bone marrow, 17.07% of the samples would have been identified as positive. In that scenario, the sensitivity of DAT-*Canis* would have been estimated as 28.57% (CI95% 13.81 to 49.96) and its specificity as 91.18% (CI95% 84.08 to 95.29). A similar pattern is observed in the accuracy of other serological tests that were challenged against the reference standard used in this study. The problem of low sensitivity in the evaluated techniques is rather concerning because the reference standard used would still be open to enhancement with the inclusion of molecular diagnostics using skin tissue, which would probably hinder the performance of the evaluated serological tests even more.^45^

In the present study, it was not possible to establish the influence of symptomatology on test accuracy because most dogs were asymptomatic and because there was no significant difference in symptom score between the infected and non-infected groups. That difference in the detection capability for CVL in symptomatic and asymptomatic dogs was explored in other studies with different serological techniques. Paltrinieri et al.^46^ mentioned that the development of sensitivity for indirect immunofluorescence in detecting CVL is, on average, 90% for symptomatic animals and 29.4% in asymptomatic animals. In the work by Santarém et al.^47^ when using ELISA rK 39, the sensitivity was 88% in symptomatic animals and 56% in asymptomatic ones. In other research, the Ministry of Health’s protocol accuracy achieved a sensitivity of 95.24 (CI95% 77.33 to 99.15) and specificity of 61.11 (CI95% 38.62 to 79.69) in symptomatic dogs. In asymptomatic dogs, the sensitivity was 51.35 (CI95% 35.89 to 66.55), and the specificity was 93.75 (CI95% 85 to 97.54).^10^

These results reinforce the concerns with using serological tests to identify asymptomatic dogs, considering that serological diagnosis is the primary surveying tool for controlling CVL. Thus, it is possible that dogs that were screened and considered seronegative by serological protocols may have been infected, and they may have remained as a reservoir for a longer time without any intervention. As stated by Lopes et al.,^7^ one in every five seronegative dogs may have been infected, highlighting the concern that has been expressed by several authors regarding the real detection capacity of the tests currently in use by control programs.^3^ ^,^ ^7^ ^,^ ^34^ ^,^ ^36^

The need for new diagnostic methods for surveys on dog populations that can discern both asymptomatic and symptomatic animals in an accurate way persists because that is a key element to defining the success or failure of CVL control.

The paradigms traditionally applied to diagnostic test validation for CVL are currently being challenged by molecular techniques and new knowledge on the role of these tests, and by their possible meanings in the context of the natural evolution of the infection or its prognosis after specific therapeutic interventions. Molecular tests are necessary to achieve a consensus on the reference standard for diagnosing the several stages of the canine disease. Attention is to be given to the results of molecular tests as infection markers for arthropod vectors in dogs because there are no easily verifiable markers for measuring that phenomenon. The molecular revolution has also affected the paradigms for diagnosing HVL. With more sensitive protocols, it is possible to diagnose patients in different stages of infection and in different types of clinical samples, including immunosuppressed patients who do not present detectable levels of antibodies in traditional serological tests.^48^ ^,^ ^49^

The actual implication of our results for controlling visceral leishmaniasis and the application of extreme control measures such as dog culling should be further researched. It seems clear that in the near future such extreme measures will be abandoned because of its low acceptance by the affected communities and the ethical issues linked to appropriate animal care. In addition, newer control measures have been under evaluation such as insecticide-impregnated dog collars and preventive or therapeutic vaccines. For those interventions maintaining infected dogs in the community, careful monitoring of infection rates will be crucial for effectiveness evaluation, and our data showed that current serological tests will fail to detect most of the infected animals without clinical signs of visceral leishmaniasis. Finally, the infectiousness of asymptomatic dogs with positive results that were revealed exclusively through parasite DNA detection assays deserves special attention until proven to the contrary, it seems reasonable to consider those dogs as potential sources of infection.

Although the endemic area where the study was done had produced a small number of HVL cases, human infection seems to be of relevant magnitude.^50^ The area has been monitored and targeted with the traditional interventions recommended by the visceral leishmaniasis control program. Moreover, it would be useful to consider that the rapid dispersion of CVL across the continent brings the need of sensitive tests for early detection of such dispersion. Currently available serological tests could be inadequate for that purpose and improving sensitivity will be crucial for that setting.

With the results from the present study, we conclude that the low sensitivity in serological tests for diagnosing asymptomatic dogs must be considered one of the top concerns for controlling visceral leishmaniasis in Brazil. It seems advisable to suggest that the concern about the lack of test specificity for diagnosing CVL may not be as relevant as the low sensitivity, because the results herein suggest that the problems in specificity from other studies may have been determined by classification errors caused by imperfect reference standards that presented low sensitivity themselves.

## References

[1] Sevá AP, Ovallos FG, Amaku M, Carrillo E, Moreno J, Galati EAB (2016). Canine-based strategies for prevention and control of visceral leishmaniasis in Brazil. PLoS One.

[2] Rocha MAN, Matos-Rocha TJ, Ribeiro CM, Abreu SR (2018). Epidemiological aspects of human and canine visceral leishmaniasis in state of Alagoas, Northeast, Brazil. Braz J Biol.

[3] Romero GAS, Boelaert M (2010). Control of visceral leishmaniasis in Latin America - A systematic review. PLoS Negl Trop Dis.

[4] Schubach EYP, Figueiredo FB, Romero GAS (2014). Accuracy and reproducibility of a rapid chromatographic immunoassay for the diagnosis of canine visceral leishmaniasis in Brazil. Trans R Soc Trop Med Hyg.

[5] Peixoto HM, Oliveira MRF, Romero GAS (2015). Serological diagnosis of canine visceral leishmaniasis in Brazil systematic review and meta-analysis. Trop Med Int Health.

[6] Larson M, Toepp A, Scott B, Kurtz M, Fowler H (2016). Semi-quantitative measurement of asymptomatic L infantum infection and symptomatic visceral leishmaniasis in dogs using Dual-Path Platform CVL. Appl Microbiol Biotechnol.

[7] Lopes EG, Sevá AP, Ferreira F, Nunes CM, Keid LB (2017). Serological and molecular diagnostic tests for canine visceral leishmaniasis in Brazilian endemic area : one out of five seronegative dogs are infected. Epidemiol Infect.

[8] Figueiredo FB, de Vasconcelos TCB, Madeira MF, Menezes RC, Maia-Elkhoury ANS, Marcelino AP (2018). Validation of Dual-path Platform chromatographic immunoassay (DPP(r) CVL rapid test) for serodiagnosis of canine visceral leishmaniasis. Mem Inst Oswaldo Cruz.

[9] Paz GF, Rugani JMN, Marcelino AP, Gontijo CMF (2018). Implications of the use of serological and molecular methods to detect infection by Leishmania spp in urban pet dogs. Acta Trop.

[10] Carvalho FLN, Riboldi EDO, Bello GL, Ramos RR, Barcellos RB, Gehlen M (2018). Canine visceral leishmaniasis diagnosis a comparative performance of serological and molecular tests in symptomatic and asymptomatic dogs. Epidemiol Infect.

[11] Solano-Gallego L, Miró G, Koutinas A, Cardoso L, Pennisi MG, Ferrer L (2011). LeishVet guidelines for the practical management of canine leishmaniosis. Parasit Vectors.

[12] Proverbio D, Spada E, Bagnagatti De Giorgi G.Perego R.Valena E (2014). Relationship between Leishmania IFAT titer and clinicopathological manifestations (clinical score) in dogs. Biomed Res Int.

[13] Gharbi M, Mhadhbi M, Rejeb A, Jaouadi K, Rouatbi M, Darghouth MA (2015). Leishmaniasis (Leishmania infantum infection) in dogs. Rev Sci Tech.

[14] Torrecilha RBP, Utsunomiya YT, Bosco AM, Almeida BF, Pereira PP, Narciso LG (2016). Correlations between peripheral parasite load and common clinical and laboratory alterations in dogs with visceral leishmaniasis. Prev Vet Med.

[15] Soares MRA, de Mendonça IL, do Bonfim JM, Rodrigues JA, Werneck GL, Costa CHN (2011). Canine visceral leishmaniasis in Teresina, Brazil relationship between clinical features and infectivity for sand flies. Acta Trop.

[16] Laurenti MD, Rossi CN, Matta VLR, Tomokane TY, Corbett CEP, Secundino NFC (2013). Asymptomatic dogs are highly competent to transmit Leishmania (Leishmania) infantum chagasi to the natural vector. Vet Parasitol.

[17] Borja LS, Sousa OMF, Solcà MS, Bastos LA, Bordoni M, Magalhães JT (2016). Parasite load in the blood and skin of dogs naturally infected by Leishmania infantum is correlated with their capacity to infect sand fly vectors. Vet Parasitol.

[18] Magalhães-Junior JT, Mota TF, Porfirio-Passos G, Larangeira DF, Franke CR, Barrouin-Melo SM (2016). Xenodiagnosis on dogs with visceral leishmaniasis canine and sand fly aspects related to the parasite transmission. Vet Parasitol.

[19] Esteva L, Vargas C, Vargas de León C (2017). The role of asymptomatics and dogs on leishmaniasis propagation. Math Biosci.

[20] Zou L, Chen J, Ruan S (2017). Modeling and analyzing the transmission dynamics of visceral leishmaniasis. Math Biosci Eng.

[21] Oliveira E, Saliba JW, Oliveira D, Dias ES, Paz GF (2016). A prototype of the direct agglutination test kit (DAT-Canis) for the serological diagnosis of canine visceral leishmaniasis. Vet Parasitol.

[22] Faria AR, Pires SF, Reis AB, Coura-Vital W, Silveira JAG, Sousa GM (2017). Canine visceral leishmaniasis follow-up a new anti-IgG serological test more sensitive than ITS-1 conventional PCR. Vet Parasitol.

[23] Dias DS, Ribeiro PAF, Salles BCS, Santos TTO, Ramos FF, Lage DP (2018). Serological diagnosis and prognostic of tegumentary and visceral leishmaniasis using a conserved Leishmania hypothetical protein. Parasitol Int.

[24] Nogueira CT, Del Cistia ML, Urbaczek AC, Jusi MMG, Velásquez AMA, Machado RZ (2018). Potential application of rLc36 protein for diagnosis of canine visceral leishmaniasis. Mem Inst Oswaldo Cruz.

[25] Paiva-Cavalcanti M, Morais RCS, Pessoa-e-Silva R, Trajano-Silva LAM, Gonçalves-de-Albuquerque SC, Tavares DHC (2015). Leishmaniases diagnosis an update on the use of immunological and molecular tools. Cell Biosci.

[26] Boelaert M, Bhattacharya S, Chappuis F, El Safi S, Mondal D, Rijal S (2007). Evaluation of rapid diagnostic tests visceral leishmaniasis. Nat Rev Microbiol.

[27] Jain NC (1986). Schalm's veterinary hematology.

[28] Romero GAS, Sampaio RNR, Macêdo VO, Marsden PD (1999). Sensitivity of a vacuum aspiratory culture technique for diagnosis of localized cutaneous leishmaniasis in an endemic area of Leishmania (Viannia) braziliensis transmission. Mem Inst Oswaldo Cruz.

[29] Ampuero J, Rios AP, Carranza-Tamayo CO, Romero GAS (2009). Genus-specific kinetoplast-DNA PCR and parasite culture for the diagnosis of localised cutaneous leishmaniasis applications for clinical trials under field conditions in Brazil. Mem Inst Oswaldo Cruz.

[30] Talmi-Frank D, Nasereddin A, Schnur LF, Schoönian G, Töz SO, Jaffe CL (2010). Detection and identification of old world Leishmania by high resolution melt analysis. PLos Negl Trop Dis.

[31] Pita-Pereira D, Lins R, Oliveira MP, Lima RB, Pereira BAS, Moreira OC (2012). SYBR Green-based Real-Time PCR targeting kinetoplast DNA can be used to discriminate between the main etiologic agents of Brazilian cutaneous and visceral leishmaniases. Parasit Vectors.

[32] MS/SVS/DVE - Ministério da Saúde/Secretaria de Vigilância em Saúde/Departamento de Vigilância Epidemiológica (2014). Manual de vigilância e controle da leishmaniose visceral.

[33] MS/SVS/DVDT - Ministério da Saúde/Secretaria de Vigilância em Saúde/Departamento de Vigilância de Doenças Transmissíveis (2011). Nota técnica conjunta 01/2011.

[34] Laurenti MD, Leandro MVS, Tomokane TY, De Lucca HRL, Aschar M, Souza CSF (2014). Comparative evaluation of the DPP CVL rapid test for canine serodiagnosis in area of visceral leishmaniasis. Vet Parasitol.

[35] Grimaldi G, Teva A, Ferreira AL, Santos CB, Pinto IS, Azevedo CT (2012). Evaluation of a novel chromatographic immunoassay based on Dual-Path Platform technology (DPP(r)CVL rapid test) for the serodiagnosis of canine visceral leishmaniasis. Trans R Soc Trop Med Hyg.

[36] Coura-Vital W, Ker HG, Roatt BM, Aguiar-Soares RDO, Leal GGDA, Moreira NDD (2014). Evaluation of change in canine diagnosis protocol adopted by the visceral leishmaniasis control program in Brazil and a new proposal for diagnosis. PLoS One.

[37] Nunes CM, de Lima VMF, Paula HB, Perri SHV, Andrade AM, Dias FEF (2008). Dog culling and replacement in an area endemic for visceral leishmaniasis in Brazil. Vet Parasitol.

[38] Mendonça IL, Batista JF, Schallig H, Cruz MSP, Alonso DP, Ribolla PEM (2017). The performance of serological tests for Leishmania infantum infection screening in dogs depends on the prevalence of the disease. Rev Inst Med Trop São Paulo.

[39] Silva RBS, Mendes RS, Santana VL, Souza HC, Ramos CPS, Souza AP (2016). Aspectos epidemiológicos da leishmaniose visceral canina na zona rural do semiárido paraibano e análise de técnicas de diagnóstico Pesq Vet. Bras.

[40] Solcà MS, Bastos LA, Guedes CES, Bordoni M, Borja LS, Larangeira DF (2014). Evaluating the accuracy of molecular diagnostic testing for canine visceral leishmaniasis using latent class analysis. PLoS One.

[41] Courtenay O, Carson C, Calvo-Bado L, Garcez R, Quinnell JR (2014). Heterogeneities in Leishmania infantum infection: using skin parasite burdens to identify highly infectious dogs.. PLoS Negl Trop Dis.

[42] Sakkas H, Garzonika C, Levidiotou S (2016). Laboratory diagnosis of visceral leishmaniasis. J Vector Borne Dis.

[43] Medeiros FAC, Gomes LI, Oliveira E, Souza CSA, Mourão MV, Cota GF (2017). Development and validation of a PCR-ELISA for the diagnosis of symptomatic and asymptomatic infection by Leishmania (Leishmania) infantum.. J Trop Med.

[44] Leal GGA, Carneiro M, Pinheiro AC, Marques LA, Ker HG, Reis AB (2018). Risk profile for Leishmania infection in dogs coming from an area of visceral leishmaniasis reemergence. Prev Vet Med.

[45] Reis LE, Coura-Vital W, Roatt BM, Bouillet LÉ, Ker HG, Fortes-de-Brito RC (2013). Molecular diagnosis of canine visceral leishmaniasis a comparative study of three methods using skin and spleen from dogs with natural Leishmania infantum infection. Vet Parasitol.

[46] Paltrinieri S, Gradoni L, Roura X, Zatelli A, Zini E (2016). Laboratory tests for diagnosing and monitoring canine leishmaniasis. Vet Clin Pathol.

[47] Santarém N, Silvestre R, Cardoso L, Schallig H, Reed SG, Cordeiro-da-Silva A (2010). Application of an improved enzyme-linked immunosorbent assay method for serological diagnosis of canine leishmaniasis. J Clin Microbiol.

[48] Hailu T, Yimer M, Mulu W, Abera B (2016). Challenges in visceral leishmaniasis control and elimination in the developing countries a review. J Vector Borne Dis.

[49] Sundar S, Rai M (2018). Laboratory diagnosis of visceral leishmaniasis. Mol Diagn Ther.

[50] Carranza-Tamayo CO, Werneck GL, Romero GAS (2016). Are opossums a relevant factor associated with asymptomatic Leishmania infection in the outskirts of the largest Brazilian cities. Braz J Infect Dis.

