# Supplementary material for: Improving the reference standard for the diagnosis of canine visceral leishmaniasis: a challenge for current and future tests
Source: Mem Inst Oswaldo Cruz. 2019 Jan 31;114:e180452. doi: 10.1590/0074-02760180452 (PMC6358009; doi:10.1590/0074-02760180452)
Supplement: Supplementary file 1 [file 1678-8060-mioc-114-e180452-s.pdf]

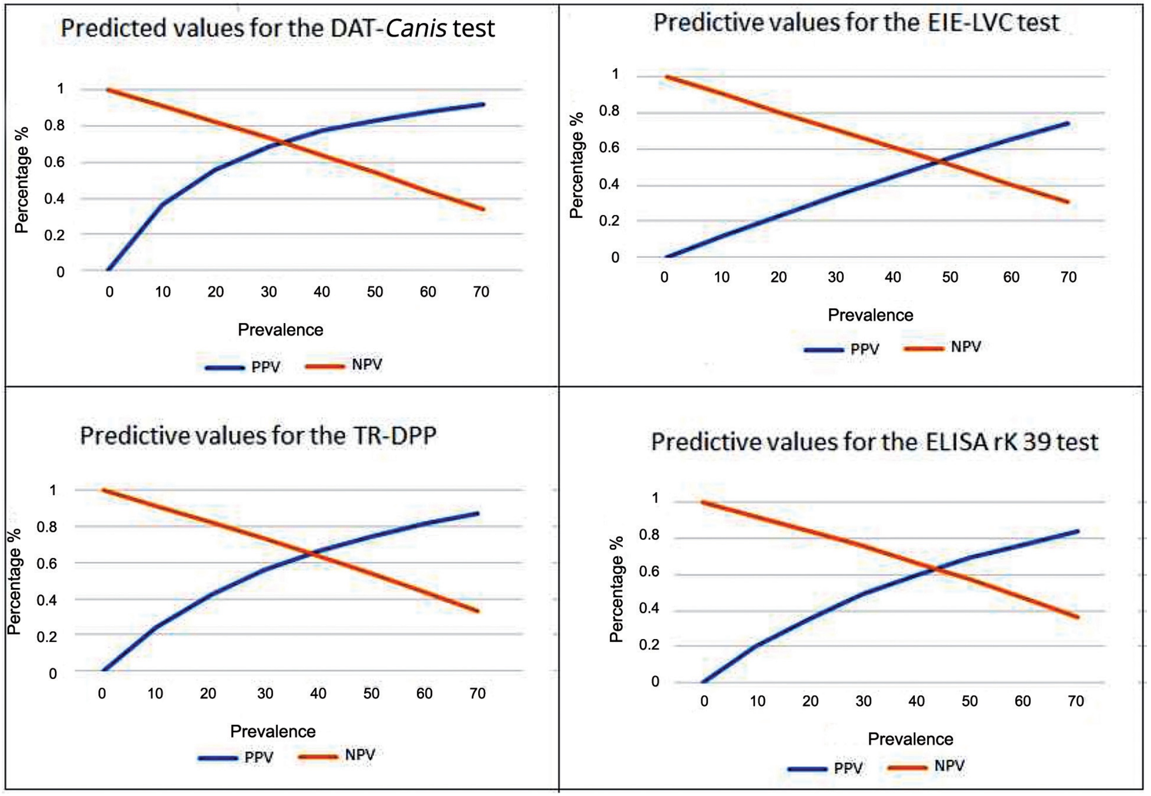

Positive and negative predictive values for the evaluated serological tests for the hypothetical prevalences of canine infection from 0 to 70%.

TABLE I

Raw agreement among the tests results that composed the reference standard. The numbers correpond to concordant positive results between each pair of tests

	Direct parasitological examination	Bone marrow culture	kDNA detection and ITS1 in peripheral blood	kDNA detection in the bone marrow	qPCR -kDNA in peripheral blood or in bone marrow
Direct parasitological examination	11	2	1	4	6
Bone marrow culture		12	1	7	9
kDNA detection and ITS1 in peripheral blood			7	2	4
kDNA detection in the bone marrow				18	14
qPCR - kDNA in peripheral blood or in bone marrow					56

CC BY

TABLE II

Raw agreement among the evaluated serological tests. The numbers correpond to concordant positive results between each pair of tests

	TR-DPP	EIE-LVC	ELISA-rK 39	DAT-*Canis*
TR-DPP[a]	19	7	11	7
EIE-LVC[b]		13	8	5
ELISA-rK 39[c]			35	10
DAT-Canis[d]				15

a: rapid test double-route platform; *b*: immuno-enzymatic assay for CVL; *c*: enzyme linked immunonosorbent assay rK 39; *d*: direct aglutination test.

TABLE III

Accuracy of serological tests in sequence testing for diagnosis of canine visceral leishmaniasis (CVL), Brasília, DF, 2017

		Reference standard		Sensitivity (CI95%)	Specificity (CI95%)	Positive likelihood ratio (CI95%)	Negative likelihood ratio (CI95%)
		Positive	Negative				
TR-DPP[a] as triage and EIE-LVC[b] as confirmation	Positive	7	0	10.14% (5.0 to 19.49)	100% (93.36 to 100)	α +	0.89 (0.83 to 0.973)
	Negative	62	54				
TR-DPP[a] as triage and ELISA rK39[c] as confirmation	Positive	9	0	13.04% (7.02 to 22.97)	100% (93.36 to 100)	α +	0.87 (0.79 to 0.95)
	Negative	60	54				
TR-DPP[a] as triage and DAT-*Canis*[d] as confirmation	Positive	7	0	10.14% (5.0 to 19.49)	100% (93.36 to 100)	α +	0.89 (0.83 to 0.97)
	Negative	62	54				
ELISA rK39[c] as triage and DAT-*Canis*[d] as confirmation	Positive	9	1	13.04% (7.02 to 22.97)	98.15% (93.36 to 100)	7.04 (0.92 to 53.90)	0.88 (0.80 to 0.97)
	Negative	60	53				
ELISA rK 39[c] as triage and TR-DPP[a] as confirmation	Positive	11	0	15.94% (9.14 to 26.33)	100% (93.36 to 100)	α +	0.84 (0.75 to 0.93)
	Negative	58	54				
EIE-LVC[b] as triage and TR-DPP[a] as confirmation	Positive	8	0	11.59% (5.99 to 21.25)	100% (93.36 to 100)	α +	0.88 (0.81 to 0.96)
	Negative	61	54				
DAT-*Canis*[d] as triage and TR-DPP[a] as confirmation	Positive	7	0	10.14% (5.0 to 19.49)	100% (93.36 to 100)	α +	0.89 (0.83 to 0.973)
	Negative	62	54				
DAT-*Canis*[d] as triage and ELISA rK 39[c] as confirmation	Positive	9	1	13.04% (7.02 to 22.97)	98.15% (93.36 to 100)	7.04 (0.92 to 53.90)	0.88 (0.80 to 0.97)
	Negative	60	53				

a: rapid test double-route platform; b: immuno-enzymatic assay for CVL; *c*: enzyme linked immunonosorbent assay rK 39; *d*: direct aglutination test.
